# Impact of Repeated Acute Exposures to Low and Moderate Exercise-Induced Hypohydration on Physiological and Subjective Responses and Endurance Performance

**DOI:** 10.3390/nu13124477

**Published:** 2021-12-15

**Authors:** Thomas A. Deshayes, Nicolas Daigle, David Jeker, Martin Lamontagne-Lacasse, Maxime Perreault-Briere, Pascale Claveau, Ivan L. Simoneau, Estelle Chamoux, Eric D. B. Goulet

**Affiliations:** 1 Faculty of Physical Activity Sciences, University of Sherbrooke, Sherbrooke, QC J1K 2R1, Canada; thomas.deshayes@usherbrooke.ca (T.A.D.); nicolas.daigle@usherbrooke.ca (N.D.); david.jeker@usherbrooke.ca (D.J.); mllacasse@yahoo.fr (M.L.-L.); maxime.perreault-briere@usherbrooke.ca (M.P.-B.); pascale.claveau2@usherbrooke.ca (P.C.); 2 Research Center on Aging, University of Sherbrooke, Sherbrooke, QC J1H 4C4, Canada; 3 Centre de Recherche et de Formation par Simulation, Cegep of Sherbrooke, Sherbrooke, QC J1E 4K1, Canada; drsimoneau1@gmail.com; 4 Faculty of Arts and Science, Biological sciences, Bishop’s University, Sherbrooke, QC J1M 1Z7, Canada; echamoux@ubishops.ca

**Keywords:** hypohydration, exercise-induced dehydration, repeated, habituation, performance

## Abstract

This study aimed to examine whether repeated exposures to low (2%) and moderate (4%) exercise-induced hypohydration may reverse the potentially deleterious effect of hypohydration on endurance performance. Using a randomized crossover protocol, ten volunteers (23 years, $\dot{V}$O_2max_: 54 mL∙kg^−1^∙min^−1^) completed two 4-week training blocks interspersed by a 5-week washout period. During one block, participants replaced all fluid losses (EUH) while in the other they were fluid restricted (DEH). Participants completed three exercise sessions per week (walking/running, 55% $\dot{V}$O_2max_, 40 °C): (1) 1 h while fluid restricted or drinking *ad libitum*, (2) until 2 and (3) 4% of body mass has been lost or replaced. During the first and the fourth week of each training block, participants completed a 12 min time-trial immediately after 2% and 4% body mass loss has been reached. Exercise duration and distance completed (14.1 ± 2.7 vs. 6.9 ± 1.5 km) during the fixed-intensity exercise bouts were greater in the 4 compared to the 2% condition (*p* < 0.01) with no difference between DEH and EUH. During the first week, heart rate, rectal temperature and perceived exertion were higher (*p* < 0.05) with DEH than EUH, and training did not change these outcomes. Exercise-induced hypohydration of 2% and 4% body mass impaired time-trial performance in a practical manner both at the start and end of the training block. In conclusion, exercise-induced hypohydration of 2% and 4% body mass impairs 12 min walking/running time-trial, and repeated exposures to these hypohydration levels cannot reverse the impairment in performance.

## 1. Introduction

Exercise-induced dehydration leading to a lower than normal body water level (i.e., hypohydration) exacerbates cardiovascular drift, core temperature strain [1,2] and glycogenolysis [3,4,5], reduces muscle [6] and cerebral blood flow [7], increases rating of perceived exertion [1,4,5] and thirst and alters mood. It has been reported that these responses are proportional to the level of hypohydration induced [1]. Individually or in concert, these factors may contribute to and explain the decrease in endurance performance associated with hypohydration, especially if exercise is performed in a hot/humid environment [8,9,10].

It has been postulated that training status, degree of heat acclimation and familiarity with hypohydration may reduce some of the physiological and performance impairments usually associated with hypohydration [11,12,13,14,15,16,17,18,19]. Endurance athletes are exposed to hypohydration on a daily basis [20]; in fact, they only replace approximately 50% of their sweat losses while consuming fluid *ad libitum* [21]. Several observational field studies have reported that important acute body mass losses accrued during endurance exercise, which can be taken as an imperfect, but nevertheless, accepted reflection of hypohydration, are sometimes associated with the best endurance performances [22,23,24,25,26,27]. Although not supportive of a cause and effect relationship, this observation nevertheless highlights the possibility that endurance athletes may, to a certain extent, adapt to hypohydration. However, few studies have experimentally investigated whether repeated acute exposures to hypohydration could, indeed, reduce physiological strain and protect subsequent performance while exercising with hypohydration. As with repeated exposures to altitude [28] or heat stress [29], two situations for which it is possible to adapt to a certain extent, it cannot be ruled out that an individual could potentially get used to the repeated effects of hypohydration, when specifically trained to tolerate its effects.

Several studies have examined whether consuming fluid or not during a heat acclimation protocol could influence performance while exercising at a single and common hydration level, before and after the heat acclimation protocol. With one exception [30], their results showed that whether one drinks or not during a heat acclimation protocol should have no consequence on exercise performance [19,31,32,33,34]. However, results of these studies preclude the understanding of whether one could adapt or not to the repeated effects of hypohydration as, to achieve so, experiments conducted while euhydrated and hypohydrated prior and after a habituation protocol are required. To this day, only one study has attempted to determine whether humans could adapt to the repeated effects of hypohydration [18]. It had active individuals train on 4 occasions in a thermoneutral climate while being hypohydrated by ~2.5% of body mass. Pre- and post-training 5 km running time-trial performance was measured while athletes were hypohydrated and euhydrated. Results showed that habituation to hypohydration significantly attenuated, but did not completely reverse, the decrease in time-trial performance. Moreover, while heart rate remained higher during the hypohydrated trial after habituation, participants reported lower perceived exertion.

Results of the Fleming and James [18] study raise several questions. Indeed, would the results have been different if (1) the habituation protocol was conducted in the heat; (2) the participants were exposed to greater levels of hypohydration during the habituation protocol and; (3) the participants underwent more habituation time/exposures. Moreover, it is unknown whether exposure to two different hypohydration levels during the habituation protocol would lead to similar or different performance effects and what kind of effect would produce habituation to a moderate hypohydration level when performance is evaluated at low hypohydration level. It cannot be excluded that certain physiological adaptations may occur with repeated hypohydration exposures and result in better physiological and perceptual responses during subsequent exposures. For example, repeated exposures to hyperosmotic stress might eventually lead to reduction in perceptual outcomes such as perceived thirst and perceived exertion, two variables that can influence endurance performance [8,13,18].

Therefore, the aim of this study was to determine the extent to which low (2%) and moderate (4% body mass) hypohydration levels impact endurance performance following exercise conducted in a hot environment while not habituated to hypohydration, and whether repeated exposures to the effect of hypohydration moderate the baseline response, compared with a situation where hypohydration is prevented during exercise. It was hypothesized that endurance performance would be significantly lowered by 2% and 4% hypohydration levels prior to habituation, that after several training sessions with repeated exposures to hypohydration the decline in endurance performance would completely be reversed at the two different hypohydration levels and that the percent change in performance with the 2% hypohydration level following habituation would be superior to that of the 4% hypohydration level.

## 2. Materials and Methods

### 2.1. Participants

Fifteen (5 women) non-heat-acclimated physically active participants were initially recruited, but ten (2 women) completed the entire study. Reasons for withdrawal included knee injury (*n* = 1), fear of hypohydration (*n* = 1), health reason unrelated to this study (*n* = 1), unable to complete the exercise session (*n* = 1) and personal reason (*n* = 1). Women were tested during the follicular phase of their menstrual cycle (period ranging from the first day of menstruation + the following 13 days). Before obtaining their informed written consent, each participant received explanations of the entire study protocol and associated risks. The CIUSSS Estrie-CHUS Ethics Committee (#2019-3081) approved all procedures. All experiments occurred from March to July in Sherbrooke, Canada.

### 2.2. Overview of This Study

**Figure 1:**
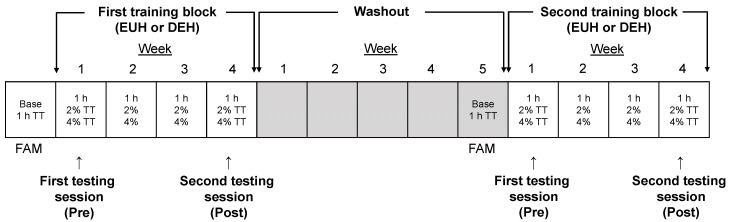
Experimental design of this study. FAM = familiarization session; Base = baseline measurements; 1 h TT = 1 h exercise + 12 min time-trial; 1 h = 1 h exercise bout only; 2% TT = exercise until a loss of 2% body mass has been lost or replaced + 12 min time-trial; 4% TT = exercise until a loss of 4% body mass has been lost or replaced + 12 min time-trial; EUH = euhydrated; DEH = dehydrated.

Figure 1 illustrates the experimental design of this study. Using a randomized and crossover protocol (for training block (euhydrated (EUH) or dehydrated (DEH)) and condition (2 or 4% body mass loss)), all participants completed two, 4-week training blocks in the heat interspersed by a 5-week washout period. The washout period aimed to eliminate the heat stress- and potential hypohydration-related adaptations that could have occurred during the first training block. The week prior to each training block participants underwent baseline measurements and were familiarized with the time-trial performance test. Each training block included 3 exercise sessions per week where participants walked/run at a fixed intensity for a 1 h period or until a body mass of 2 or 4% had been lost (DEH) or replaced (EUH). Endurance performance assessments were completed during the testing sessions that occurred the first and fourth week of each training block, immediately following the 2% and 4% fixed-intensity exercise bouts.

### 2.3. Baseline Measurements

During their first visit to the laboratory, participants underwent measurements of their post-void body mass with a floor scale (±20 g, BX-300+, Atron Systems, West Caldwell, NJ, USA), height with a wall stadiometer, resting blood pressure and heart rate after a 3 min rest period with a digital sphygmomanometer (Welch Allyn 420 series, Skaneateles Falls, NY, USA) and body composition with dual-energy X-ray absorptiometry (Lunar Prodigy, GE Healthcare, Chicago, IL, USA). Maximal oxygen consumption ($\dot{V}$O_2max_) was measured on a motorized treadmill (model TMX428 Trackmaster, Newton, KS, USA) using an expired gas analysis system (Cosmed Quark CPET, Cosmed, Chicago, IL, USA) that had been calibrated with gases of known concentration. The attainment of $\dot{V}$O_2max_ was confirmed using the ACSM criteria [35].

### 2.4. Familiarization Session

The aim of this trial consisting in a 1 h exercise session followed by a time-trial was to determine the best combination of speed and grade to be used by participants during the remainder of the training block and familiarize themselves with the 12 min time-trial. After having void, participants entered the environmental chamber (maintained at 40 °C and 20–30% relative humidity) and were instrumented with a face mask to measure $\dot{V}$O_2_. Participants then began exercising and were told to adjust the treadmill’s grades and speeds such to find the best combination eliciting 55% $\dot{V}$O_2max_. Once this was achieved and $\dot{V}$O_2_ had stabilized, the face mask removed. This entire process took 20–30 min. Immediately following the 1 h exercise bout, participants completed a 12 min self-paced time-trial, at the pre-selected grade. During the EUH training block participants could drink water *ad libitum* during the fixed-intensity exercise period, whereas during the DEH training block they were deprived from fluid consumption.

### 2.5. Pre-Experimental Procedures

Food and fluid intakes were recorded for 24 h prior to the 2% and 4% exercise sessions of the first week of each training block. Then, participants were required to replicate food and fluid intake before all subsequent 2% and 4% exercise sessions. No such control occurred before the 1 h exercise session. Sports supplements and physical activity were restricted, respectively, for the last 24 and 12 h before each exercise session. Prior to each exercise session participants were required to consume 250 mL of water 1 h before bedtime. The same amount of water was also consumed 1 h before arrival at the laboratory, after which participants were asked to remain fast. No other instructions were provided to participants between exercise sessions.

### 2.6. Exercise/Testing Sessions

On their arrival at the laboratory, participants emptied their bladder, collected a urine sample, were nude weighted, dressed themselves, installed a chest electrode and rectal probe and, only for the testing sessions, were instrumented with a catheter. Participants then entered the environmental chamber (40 °C and 20–30% relative humidity, wind speed: ~5 km∙h^−1^), rested on the treadmill for 2 min (or 10 min for the testing sessions) and then started exercise, which consisted of walking or light running at the self-selected speed and grade chosen during the familiarization trial.

The first weekly exercise session always consisted in the completion of 2 × 30 min exercise bouts interspersed by a recovery period of 3 min inside the chamber. During this session, participants could (EUH) or could not (DEH) drink water *ad libitum*. Over the next 2 exercise sessions, participants completed cycles consisting of 8 min of exercise followed by 2 min of recovery inside the chamber until a sweat-induced body mass loss of either 2 or 4% was reached. During each of the 2 min recovery period subjects were requested to urinate and then were weighted. Urine losses were not considered in the cumulated loss of body mass and hence were entirely replaced during exercise. Sweat-induced body mass loss was computed with the following formula:

SIBML = BM of the preceding weighing period (kg) − BM of the current weighing period (kg) + WC during the exercise period immediately preceding the weighing period (kg) − UP during the exercise period immediately preceding the weighing period (kg)(1)

SIBML is sweat-induced body mass loss;

BM is body mass;

WC is water consumed and;

UP is urine produced.

With EUH, participants were requested to consume water at whenever time they wanted over a period of 8 min at an amount corresponding to the sweat and urine losses cumulated over the prior 10 min period. With DEH, participants were fluid restricted but were allowed to rinse their mouths with water at any time. Moreover, they could pour cold water on their bodies and heads at a rate of 100 mL∙h^−1^ (both EUH and DEH). During these 2 exercise sessions, $\dot{V}$O_2_ was continuously measured during the first 10 min of exercise and treadmill speed adjusted accordingly to elicit the required intensity. On the first and fourth week of both training blocks (i.e., testing sessions), participants were requested to complete a performance test immediately following the achievement of the targeted body mass loss (i.e., 2 or 4%). The test consisted of a self-paced 12 min time-trial where participants had to cover the greatest distance possible [36] at the same grade as during the fixed-intensity period (mean: 5 ± 4%, min-max: 1–10%). Participants were given access to performance time but not speed. Participants always exercised with the same gears and testing sessions were performed at the same time of the day. Following 1 h of exercise and every 30 min thereafter, participants were requested to consume energy gels (GU Energy gel, Berkeley, CA, USA) containing 22 g of carbohydrates and 60 mg of sodium.

### 2.7. Measurements

Heart rate was measured with a Garmin chest electrode (USB ANT stick (Garmin, Olathe, Kansas, USA) + Golden Cheetah software) and rectal temperature using the reusable telemetric pill technique (CoreTemp, HQ, Palmetto, FL, USA) [37]. Rectal temperature, perceived exertion (15-point Borg scale (from 6–20); [38]), thirst (11-point scale; [39]) and thermal comfort (7-point scale; [40]) were measured every 4 min. During the self-paced 12 min time-trial, all the variables of interest were collected at min 4, 8 and 12. Percent dehydration was computed as the difference between pre- and post-exercise body mass (post-void) relative to pre-exercise body mass. Sweat loss was calculated by subtracting the post- from the pre-exercise body mass (post-void) while correcting for fluid intake and urine losses. Whole-body sweat rate was determined by dividing sweat loss by total exercise duration (fixed-intensity + 12 min time-trial). Respiratory water losses and losses of mass associated with the respiratory exchange of O_2_ and CO_2_ were not considered and assumed to be similar among trials.

### 2.8. Blood Measures

Only during the testing sessions, a flexible catheter was inserted in the antecubital vein and venous blood samples (12 mL) were taken at rest (after the 10 min stabilization phase in the chamber on the treadmill), at ~50% of the total exercise time and at the end of the fixed-intensity exercise bout. The blood was collected in either heparinized or EDTA tubes. Hemoglobin was analyzed by spectrophotometry (Alere H2 Hemopoint, Waltham, MA, USA) and hematocrit by microcentrifugation (5 min at 10,000× *g*). Heparinized blood was centrifuged at room temperature at 2000× *g* for 5 min and the extracted plasma was stored at −20 °C until analyses. Blood collected in EDTA tubes was centrifuged (2000× *g*, 10 min) at 4 °C and plasma was stored at −80 °C until analyses. Plasma volume changes were computed accorded to Dill and Costill [41], and plasma natremia was measured using the ion selective electrodes technique (Easy Electrolytes, Medica Corporation, Bedford, MA, USA), plasma osmolality the freezing point depression technique (Micro Osmometer, Osmette, Precision Systems Inc., Natick, MA, USA) and plasma aldosterone enzyme-linked immunosorbent assay commercially available kits (Cayman Chemical Company, Ann Arbor, MI, USA). Intra- and inter-assay coefficients of variation (CV) were, respectively, 8.6% and 6.9%).

### 2.9. Statistical Analyses

All statistical analyses were performed using IBM SPSS Statistics software (version 26, New York, NY, USA). Shapiro–Wilk tests were used to analyze data normality. Dependent t-tests or Wilcoxon *signed-rank tests* were used to compare the characteristics of the participants and to verify whether physiological changes were observed between the first and the second training block. Two and three-factor (hydration*condition*training) repeated-measures ANOVAs and, for variables with missing cases, linear mixed models were used to determine the impact of, and interaction effects between, hydration (EUH vs. DEH), condition (2 vs. 4%) and training (pre (i.e., first testing session) vs. post (i.e., second testing session)). In cases where sphericity was violated, Greenhouse–Geisser correction was applied. When significant interaction effects were detected, multiple pairwise comparisons were performed and corrected with the false discovery rate procedure. Because of inter- and intra-participants differences in exercise duration, data collected at several time points are reported based on percentage of total exercise duration. Data are presented as the means ± standard deviations (SD), with 95% confidence intervals (95% CI) for endurance performance only. Statistical significance was set at *p* ≤ 0.05. All presented data are *n* = 10 unless stated otherwise.

The distance completed during the 12 min self-paced time-trial test was used as the dependent variable for the sample size calculation. Based on a counterbalanced protocol, a two-tailed t-test, a test–retest CV estimated at around 1.7% [36], a coefficient α of 0.05, a power of 80% and a desired minimum difference between EUH and DEH of 3.5% (twice the test–retest CV), the sample size required to detect a significant difference between conditions was of at least 7 participants.

## 3. Results

### 3.1. Participants’ Characteristics before Starting Both Training Block

Table 1 shows participants’ characteristics prior to starting both training blocks. Participants started both training blocks with similar cardiorespiratory fitness and body composition levels (all *p* > 0.05). Moreover, mean heart rate, rectal temperature and whole-body sweat rate maintained during the first 1 h exercise bout, as well as the 12 min time-trial time during the familiarization session, were similar during the first (133 ± 8 beats∙min^−1^, 38.3 ± 0.4 °C, 1.3 ± 0.3 L∙h^−1^, and 2.00 ± 0.67 km, respectively) and the second (133 ± 8 beats∙min^−1^, 38.3 ± 0.3 °C, 1.3 ± 0.3 L∙h^−1^, and 1.95 ± 0.57 km, respectively) training block (all *p* > 0.05), suggesting that no training or acclimation effects were present at the beginning of the second training block.

### 3.2. Environmental Conditions during the Testing Sessions

Environmental conditions (40.0 ± 0.2 °C and 25.7 ± 2.1% of relative humidity) remained constant through all testing sessions (all *p* > 0.05), except for a condition effect for relative humidity (Δ = 1.2%, *p* = 0.003).

### 3.3. Hydration State Prior to the Testing Sessions

Participants started all testing sessions in a similar hydration state, as indicated by the constant pre-exercise body mass, hematocrit, heart rate and plasma osmolality across trials (Table 2, all *p* > 0.05).

### 3.4. Hydration State during the Testing Sessions

**Figure 2:**
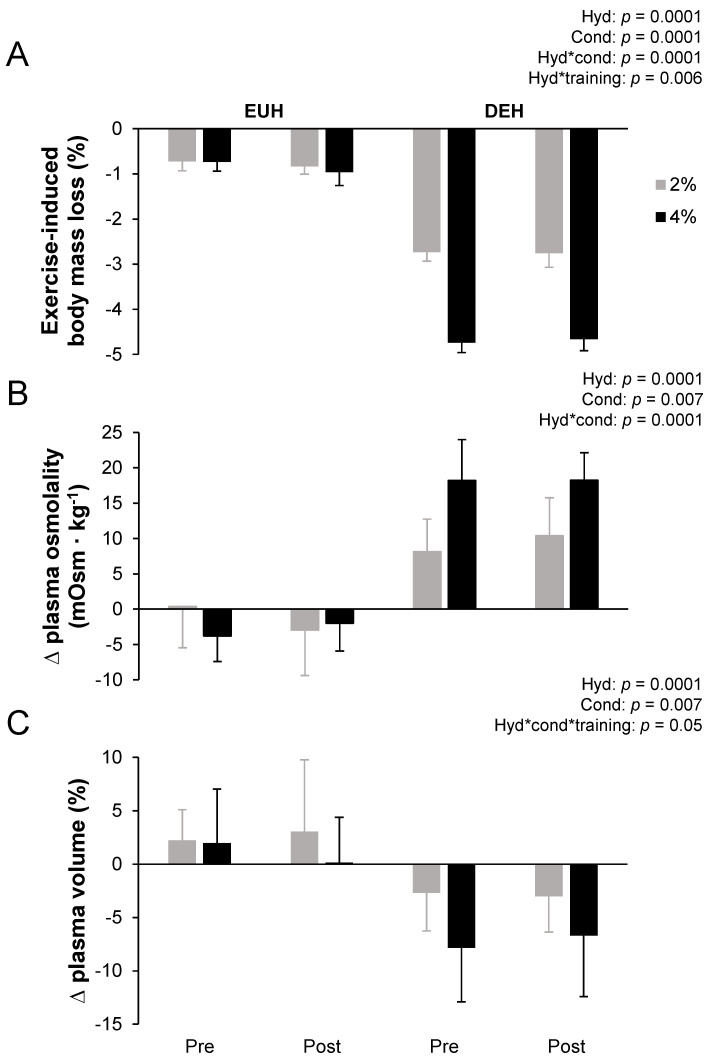
Exercise-induced body mass losses at the end of the 12 min time-trial (**A**) and changes in plasma osmolality (**B**) and plasma volume (**C**) measured at the end of the fixed-intensity exercise periods while replacing or not 2 or 4% body mass losses, pre- and post-training. Δ = difference. Values are the means ± SD. EUH = euhydrated; DEH = dehydrated; Cond = condition (2 vs. 4%); Hyd = hydration (EUH vs. DEH); Training = pre vs. post. Only the significant effects are reported.

Figure 2 depicts the % body mass losses measured at the end of the 12 min time-trial (a) and changes in plasma osmolality (b) and plasma volume (c) measured at the end of the fixed-intensity exercise period during the first and second testing sessions after the replacement or loss of 2% and 4% body mass. As to be expected, exercise-induced body mass loss at the end of the 12 min time-trial was higher with DEH, with an average of 3.7 ± 1.0%, while it remained < 1% (0.8 ± 0.2%) with EUH. There were condition, hydration*condition and hydration*training effects, but no other main effects (all *p* > 0.05). Post hoc analyzes revealed that the condition effect was observed only with DEH (*p* = 0.0002), but not with EUH (*p* = 0.10) and that the training effect was only observed with EUH (*p* = 0.005) but not with DEH (*p* = 0.48).

At the end of the fixed-intensity exercise bout plasma osmolality increased more with DEH than with EUH (13.7 ± 2.6 vs. −2.1 ± 2.7 mOsm∙kg^−1^), reaching 301.3 ± 6.7 vs. 287.4 ± 4.9 mOsm∙kg^−1^, respectively, with condition and hydration*condition effects (all *p* < 0.05). Post hoc analyzes revealed that the hydration effect was present in both conditions and that the condition effect was observed only with DEH (2%: 9.3 ± 3.2 vs. 4%: 18.2 ± 3.4 mOsm∙kg^−1^, *p* = 0.0002) but not with EUH (2%: −1.3 ± 4.3 vs. 4%: −2.9 ± 2.9 mOsm∙kg^−1^, *p* = 0.35). There were also no differences in the extent of the plasma osmolality changes between the first and the second testing session as suggested by the absence of training effect (*p* = 0.86).

Plasma volume at the end of the fixed-intensity exercise bout was significantly lower with DEH than with EUH, with condition and hydration*condition*training effects (all *p* < 0.05). However, there were no differences in the extent of the plasma volume changes between the first and the second testing session. There was an absence of training effect (*p* = 0.95), as well as no hydration*condition (*p* = 0.22), hydration*training (*p* = 0.59) or condition*training (*p* = 0.66) effects. Post hoc analyzes revealed that the condition effect was only observed with DEH pre (DEH post: *p* = 0.054) and that the hydration effect was only present for 2% pre, 4% pre and 4% post (all *p* < 0.01, 2% post: *p* = 0.062). Main effects of condition (Δ = 0.1 L∙h^−1^, *p* = 0.001) and training (increase of ~ 0.1 L∙h^−1^, *p* = 0.0001) were observed for whole-body sweat rate. However, no hydration (*p* = 0.39) or other interaction effects were observed (all *p* > 0.05).

Pre-exercise plasma aldosterone concentrations were similar across all testing sessions (200 ± 109 pg∙mL^−1^, all *p* > 0.05). However, at the end of the preload, plasma aldosterone concentrations were higher with the 4 than the 2% condition (*p* = 0.003), with hydration*condition (*p* = 0.01) but no hydration (*p* = 0.07), training (*p* = 0.08), condition*training (*p* = 0.22), hydration*training (*p* = 0.19) or hydration*condition*training (*p* = 0.054) effects. Post hoc analyzes revealed that condition effect was present for both, DEH (*p* = 0.02) and EUH (*p* = 0.04), but that the hydration effect was only present for the 4 (+140 ± 166 pg∙mL^−1^, *p* = 0.03) but not the 2% condition (+25 ± 136 pg∙mL^−1^, *p* = 0.57).

### 3.5. Exercise Duration, Distance and Intensity during the Fixed-Intensity Exercise Bout during the Testing Sessions

Exercise duration (129.4 ± 16.9 vs. 63.8 ± 9.5 min) and distance completed (14.1 ± 2.7 vs. 6.9 ± 1.5 km) during the fixed-intensity exercise bout were greater with the 4 than the 2% condition (both *p* = 0.0001). Both decreased through training (*p* = 0.0001 and 0.002) with a condition*training effect (*p* = 0.02 and 0.04). Post hoc analyzes revealed that exercise duration decreased with both conditions (2%: −3.5 ± 3.7 min, *p* = 0.01 and 4%: −10.8 ± 7.3 min, *p* = 0.002) whereas distance completed only decreased with the 4 (−0.8 ± 0.7 km, *p* = 0.007) but not with the 2% condition (−0.2 ± 0.4 km, *p* = 0.11). No hydration (*p* = 0.45 and 0.86), hydration*condition (*p* = 0.08 and 0.14), hydration*training (*p* = 0.07 and 0.13) or hydration*condition*training (*p* = 0.15 and 0.13) effects were observed. Overall, mean exercise intensity was 56 ± 2% $\dot{V}$O_2max_ (all *p* > 0.05).

### 3.6. Physiological and Subjective Variables

#### 3.6.1. Fixed-Intensity Exercise

**Figure 3:**
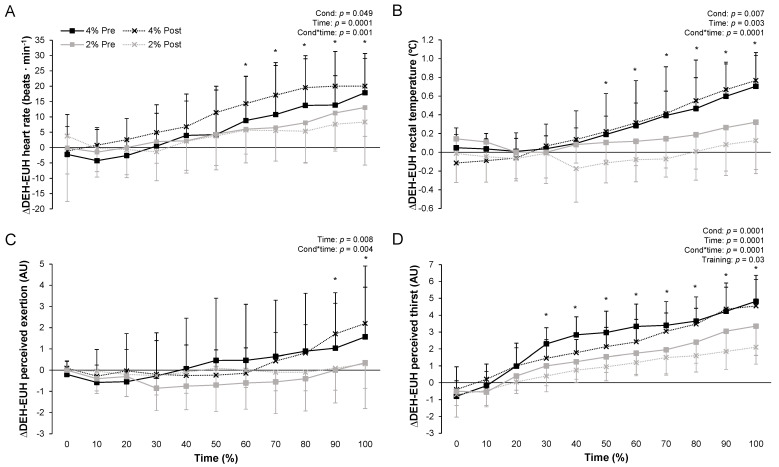
Differences between DEH and EUH during the fixed-intensity exercise periods for heart rate (**A**), rectal temperature (**B**), perceived exertion (**C**) and perceived thirst (**D**) through exercise time, while replacing or not 2 or 4% body mass losses, pre- and post-training. Δ = difference. Values are the means ± SD. EUH = euhydrated; DEH = dehydrated; Cond = condition (2 vs. 4%); Training = pre vs. post; Time = % of the fixed-intensity exercise completed. Only the significant effects are reported. * *p* < 0.05 2 vs. 4%.

Figure 3 depicts heart rate (a), rectal temperature (b), perceived exertion (c) and perceived thirst (d) differences between DEH and EUH through time, pre- and post-training while replacing or not 2 or 4% body mass. Heart rate, rectal temperature and perceived exertion followed the same pattern with difference between DEH and EUH that increased through time (all *p* < 0.01), with condition (*p* = 0.049 and 0.007 but 0.11 for perceived exertion) and condition*time (all *p* < 0.01) effects. Post hoc analyzes revealed that the difference was higher with the 4 compared to the 2% condition from 60% (heart rate), 50% (rectal temperature) and 90% (perceived exertion) of the time onward. However, the negative impact of DEH upon heart rate, rectal temperature and perceived exertion remained constant even after repeated exposures as suggested by the absence of training effect (*p* = 0.57, 0.22 and 0.36). No other effects were observed (all *p* > 0.05). At the end of the fixed-intensity exercise bout mean heart rate difference between DEH and EUH reached 11 ± 9 and 19 ± 8 beats∙min^−1^ (both *p* < 0.01) for the 2% and 4% conditions, respectively. For rectal temperature and perceived exertion, these values reached 0.2 ± 0.4 (*p* = 0.10) and 0.7 ± 0.3 °C (*p* < 0.01) and 0 ± 1 (*p* = 0.46) and 2 ± 2 arbitrary units (*p* = 0.03), respectively.

Main effects of condition and time as well as condition*time were observed for mean difference between DEH and EUH for perceived thirst (all *p* < 0.01). Post hoc analyzes revealed that the difference was higher in the 4 compared to the 2% condition from 30% of the time onward. Interestingly, the negative impact of DEH upon thirst decreased through repeated exposures as suggested by the significant training effect (−0.5 ± 0.6 arbitrary unit, *p* = 0.03). However, this decrease was similar between conditions as suggested by the absence of a condition*training effect (*p* = 0.49). No other effects were observed (all *p* > 0.05). At the end of the fixed-intensity exercise bout mean difference between DEH and EUH reached 3 ± 1 and 5 ± 1 arbitrary units (both *p* < 0.01) for the 2% and 4% conditions.

#### 3.6.2. The 12 min Time-Trial

Mean heart rate maintained during the time-trial was higher with DEH compared to EUH (181 ± 10 vs. 177 ± 16 beats∙min^−1^, *p* = 0.045) with condition (*p* = 0.051) but no training (*p* = 0.16) or other interaction effects (all *p* > 0.05). Mean rectal temperature was higher with DEH compared to EUH (39.5 ± 0.5 vs. 39.0 ± 0.4 °C, *p* = 0.0001) with condition (*p* = 0.0001), training (decrease of 0.1 ± 0.3 °C, *p* = 0.02) and hydration*condition (*p* = 0.0001) effects. Post hoc analyzes revealed that values were higher with DEH compared to EUH (ΔDEH-EUH: 2%: 0.2 ± 0.5 and 4%: 0.7 ± 0.3 °C), with higher values at 4% in both, EUH and DEH (all *p* < 0.05).

Mean perceived exertion and thirst followed the same pattern, with higher values in DEH compared to EUH (perceived exertion: 16 ± 2 vs. 15 ± 2 arbitrary units, *p* = 0.02 and thirst: 8 ± 2 vs. 4 ± 2 arbitrary units, *p* = 0.0001), with condition (*p* = 0.01 and 0.002) and hydration*condition (*p* = 0.007 and 0.0001), but no training (*p* = 0.26 and 0.66), hydration*training (*p* = 0.32 and 0.38), condition*training (*p* = 0.65 and 0.47) or hydration*condition*training (*p* = 0.52 and 0.07) effects. Post hoc analyzes revealed that perceived exertion was only higher with 4% DEH (*p* = 0.004) but not 2% DEH (*p* = 0.23) compared to EUH, and that no condition effect was present in EUH (*p* = 0.15). Perceived thirst was higher with DEH compared to EUH at 2% and 4% (*p* < 0.01), without condition effect with EUH (*p* = 0.13).

### 3.7. Endurance Performance

**Figure 4:**
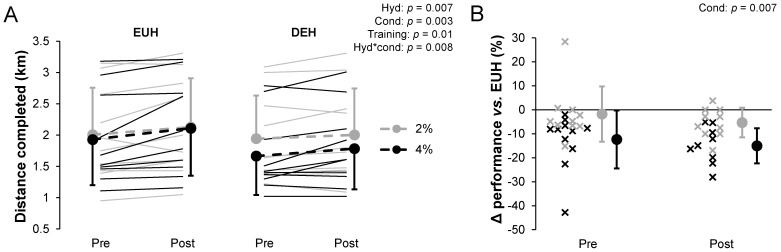
Distance completed during the 12 min time-trial (**A**) and percent performance difference (**B**) while replacing or not 2 or 4% body mass losses, pre- and post-training. Δ = difference. Values are the means ± SD. EUH = euhydrated; DEH = dehydrated; Cond = condition (2 vs. 4%); Hyd = hydration (EUH vs. DEH); Training = pre vs. post. Only the significant effects are reported.

The total mean distance completed was 2.01 ± 0.75 (EUH pre), 1.94 ± 0.69 (DEH pre), 2.13 ± 0.78 (EUH post), and 2.01 ± 0.74 km (DEH post) for the 2% condition and 1.93 ± 0.73, 1.66 ± 0.62, 2.11 ± 0.76, and 1.79 ± 0.65 km, respectively, for the 4% condition (Figure 4A). There were hydration, condition, training, and hydration*condition effects (all *p* < 0.05). Post hoc analyzes revealed that the distance completed was significantly lower in 4% DEH compared to 4% EUH (−0.30 ± 0.27 km, *p* = 0.01, −13.6%, 95% CI [−20.1 to −7.2%]) but not with 2% DEH compared to 2% EUH (−0.09 ± 0.15 km, *p* = 0.052, −3.5%, 95% CI [−9.0 to 1.9%]). Condition effect was only present for DEH (*p* = 0.008) but not EUH (*p* = 0.21). No hydration*condition*training effect was observed (*p* = 0.98). The distance completed increased similarly throughout training for DEH and EUH for the 2 (EUH: 0.12 ± 0.18 and DEH: 0.06 ± 0.13 km) and the 4% condition (EUH: 0.18 ± 0.21 and DEH: 0.12 ± 0.18 km) as suggested by the absence of condition*training (*p* = 0.25) and hydration*training (*p* = 0.16) effects (Figure 4A). The distance completed was 1.8 ± 11.5% (95% CI: −10 to 6.5%) lower during the 2% DEH pre than the 2% EUH pre and 5.3 ± 6.1% (95% CI: −9.7 to −0.9%) lower in the 2% DEH post compared to the 2% EUH post. For the 4% condition, these values reached 12.3 ± 12.1% (95% CI: −20.9 to −3.7%) and 15.0 ± 7.3% (95% CI: −20.2 to −9.7%), respectively, with condition (*p* = 0.007) but not training (*p* = 0.18) or condition*training (*p* = 0.83) effects (Figure 4B).

## 4. Discussion

The aims of this study were to (1) compare the impact of low (i.e., 2% body mass) and moderate (i.e., 4% body mass) exercise-induced hypohydration to euhydration on endurance performance while not being accustomed to hypohydration and later, after several training sessions; (2) verify the impact that repeated acute exposures to these exercise-induced hypohydration levels have on endurance performance, compared with repeated exposures to euhydration. Our results showed that, prior to habituation to the effect of hypohydration, low and moderate exercise-induced hypohydration impairs endurance performance in a practical manner comparatively to a situation where euhydration is maintained, and that following habituation to exercise-induced hypohydration, the observable improvement in endurance performance is independent of whether one trains while being euhydrated or to tolerate low and moderate hypohydration levels.

### 4.1. Impact of Low and Moderate Exercise-Induced Hypohydration Levels While Not Trained to Tolerate Their Effects

Both, hypohydration-induced physiological and endurance performance effects have been well studied. While this has been challenged [42,43], it is frequently argued that exercise-induced hypohydration ≥ 2% of body mass impairs endurance performance [8,44,45]. Hence, it is unsurprising that our study observed an hypohydration level-related effect, with a larger reduction in endurance performance (~14%) at 4 compared to 2% of body mass loss. While from a pure statistical point of view, endurance performance was not significantly reduced by the 2% of body mass loss, from a practical point of view, the 3.5% reduction in time-trial performance is unlikely to be trivial, as it corresponds to twice the CV of our performance test [36]. Therefore, our data support the claim that, under the current exercise scenario, hypohydration ≥ 2% of body mass impairs endurance performance in a worthwhile manner [8,44,45].

At the end of the fixed-intensity exercise bout, just before the time-trial commencement, plasma volume had decreased and plasma osmolality increased in an hypohydration level-dependent fashion, which is in line with results previously observed during cycling in the heat [1] and in a thermoneutral climate [46]. However, although the impact of hypohydration upon perceived thirst and perceived exertion as well as cardiovascular and thermal drifts were higher with the 4% condition, compared with the 4% euhydration condition, rectal temperature and perceived exertion at the end of the fixed-intensity exercise were not exacerbated by the 2% hypohydration condition. Such an observation has been previously reported [1,47,48] and could partially explain the larger reduction in endurance performance observed with the 4 than the 2% body mass loss condition.

### 4.2. Impact of Repeated Exposures to Low and Moderate Exercise-Induced Hypohydration Levels

In the current study, we observed that despite the distance completed during the 12 min time-trial was lower with DEH, it increased relatively similarly through training within the 2% and 4% hydration conditions and this, both with EUH and DEH. This indicates that a training effect was responsible for the improvement in time-trial performance during post-testing and that consequently no adaptations to hypohydration developed with DEH that participants may have specifically benefited from during the training block conducted in a hypohydration state. Moreover, it implies that deliberate repeated exposures to hypohydration over time during exercise should not compromise the ability to benefit from the adaptations necessary to improve time-trial performance. Finally, our results indicate that undergoing repeated exposures to exercise-induced moderate levels of hypohydration is unlikely to provide any performance advantage during exercise conducted at a low hypohydration level.

Several studies [19,31,32,33,34] have investigated the impact of training while replacing or not fluid during a heat acclimation protocol and their findings generally show that the training-induced improvement in exercise performance is independent of the hydration status maintained during heat acclimation. These findings cannot be interpreted to suggest that training-induced habituation to hypohydration is, or is not possible, as baseline and post acclimation differences in exercise performance while euhydrated and hypohydrated were not assessed.

Contrary to Fleming and James [18], our results do not support the idea that habituation to hypohydration attenuates the initial decline in performance associated with being unfamiliar to a particular hypohydration level. Our observation is nevertheless similar to the one made by Adolph [11] who concluded more than 70 years ago that exercise performance does not respond more favorably after than before repeated exposures to hypohydration levels of 5–6% of body mass. Why our results differ from those of Fleming and James [18] can possibly be attributed to methodological differences. Our participants exercised at 40 °C whereas those in Fleming and James [18] study exercised at 22 °C. It is not impossible that habituation to hypohydration may develop more easily during training conditions associated with less physiological strain or be easier to detect during time-trials conducted in a thermoneutral climate. Between pre- and post-testing sessions our subjects were trained to tolerate hypohydration for 624 (i.e., 10 h), compared to 240 min (i.e., 4 h) for Fleming and James [18]. Maybe that habituation to hypohydration develops only over a precise temporal scale shorter than the one we tested. Fleming and James [18] used a combination of 24 h of fluid deprivation followed by a bout of exercise without fluid access to manipulate hypohydration state. It cannot be discarded that the time spent hypohydrated outside the training periods with low fluid intake is a major catalyst of the training-induced habituation to hypohydration. Notwithstanding the observations by Fleming and James [18], it is important to note that our study included a control, euhydrated group. If the goal is to understand whether humans can adapt to exercise-induced hypohydration while repeatedly exposed to its effect, this is essential. In fact, with the Fleming and James [18] protocol, it cannot be discerned clearly whether the impact on exercise performance is due to the passing of time (training effect) or related to an habituation to hypohydration. Indeed, the possibility that Fleming and James [18] would have come to the same observation had their subjects trained only with euhydration cannot be discarded.

The absence of an habituation effect to repeated training-induced hypohydration exposures upon endurance performance correlates with the lack of effect we observed upon plasma volume, plasma osmolality, rectal temperature, heart rate and perceived exertion. Contrary to Fleming and James [18], we did not observe any attenuation of the negative impact of hypohydration upon perceived exertion. This could potentially explain why we did not observe an attenuation in hypohydration-induced decrements in performance after repeated exposures to hypohydration. In fact, perceived exertion is a pivotal component of endurance performance [49,50]. Perceived thirst is the only outcome that has been observed to be reduced after repeated exposures to hypohydration. This is the first study to report such an observation in the current research context; however, a recent study has reported a moderate reduction in perceived thirst after heat acclimation with *ad libitum* fluid intake [51]. Surprisingly, perceived thirst was reduced while the magnitude of change in plasma volume and plasma osmolality during exercise remained similar through training. However, such findings need to be interpreted carefully given that the magnitude of the reduction in thirst (0.5 arbitrary units) was low and, therefore, of little practical importance.

The results of the present study must be interpreted keeping the following limitations in mind. First, the 12 min time-trial was performed immediately after the fixed-intensity exercise bout aiming to manipulate the participants’ hydration state. Training times to achieve 2% and 4% hypohydration were lower at post- than pre-testing. Therefore, it cannot be excluded that this may have contributed to improve time-trial performance post-testing. Certainly, however, this design increases the ecological validity of our findings. Our results apply only to non-heat-acclimated physically active participants, mainly men, training in hot temperatures. Whether the current results apply equally to women is unknown. Indeed, some evidence suggests that sex-based differences exist in hypohydration-induced thermoregulatory and cardiac strain [52]. In the present study, the menstrual phase was estimated via self-report. This may not have been adequate for all women given the variability in the menstrual cycle and the lack of confirmation via serum measures of estrogen and progesterone. However, (1) the menstrual cycle phase seems to have no or trivial effect on endurance performance [53,54] but (2) may impact internal temperature differently [55]. As the testing sessions always occurred at the same time of the menstrual cycle, these factors are unlikely to have had any influence on our observations. Outside the testing periods, participants were trained to habituate to hypohydration for only a period of 2 weeks, or 8 training sessions (total of 624 min). Whether a shorter or longer training time would change the outcome is unknown.

## 5. Conclusions

Our results show that (1) acute exposures to low and moderate exercise-induced hypohydration while untrained to tolerate the hypohydration effect impairs endurance performance in the heat and; (2) these effects do not appear to be diminished nor exacerbated after 624 min (i.e., 10 h) or a total of 8 repeated exposures to low and moderate dehydration. These results have implications not only for athletes but also for individuals frequently called upon to move to and perform in hot environments where access to water is restricted, predisposing them to hypohydration. More specifically, they suggest that individuals who repeatedly expose themselves to low and moderate hypohydration levels should not expect an attenuation of the detrimental effects of dehydration on their performance.

## Figures and Tables

**Table 1:** Participants’ characteristics before starting each training block.

Characteristics	Before First Training Block	Before Second Training Block	*p*-Value
Age (years)	23 ± 5	23 ± 5	0.32
Height (cm)	176 ± 8	176 ± 8	0.68
Body mass (kg)	71.6 ± 11.8	71.2 ± 12.2	0.30
Body mass index (kg·m^−2^)	23.1 ± 2.9	22.9 ± 3.1	0.26
Relative maximal oxygen consumption (mL∙kg^−1^∙min^−1^)	55 ± 7	54 ± 7	0.25
Absolute maximal oxygen consumption (mL∙min^−1^)	3925 ± 861	3865 ± 809	0.36
Maximal heart rate (beats∙min^−1^)	193 ± 7	191 ± 7	0.12
Fat-free mass (%)	82.4 ± 6.5	82.3 ± 5.7	0.81
Fat mass (%)	14.0 ± 6.6	14.2 ± 5.8	0.72

Date are the means ± SD. All data are *n* = 10 except for fat-free mass and fat mass (both *n* = 9).

**Table 2:** Hydration state prior to each testing session with euhydration and dehydration.

Variables	First Testing Session		Second Testing Session	
	2% Condition	4% Condition	2% Condition	4% Condition
	Euhydrated			
Body mass (kg)	71.6 ± 12.5	71.4 ± 12.3	71.2 ± 11.7	71.1 ± 11.6
Hematocrit (%)	44.5 ± 3.0	44.8 ± 2.2	44.1 ± 3.6	44.3 ± 3.1
Heart rate (beats∙min^−1^)	87 ± 17	87 ± 16	82 ± 10	85 ± 14
Plasma osmolality (mOsm∙kg^−1^)	287.2 ± 4.8	289.0 ± 3.7	292.0 ± 5.4	289.9 ± 5.0
Plasma aldosterone (pg∙mL^−1^)	205 ± 102	166 ± 82	183 ± 102	190 ± 102
	Dehydrated			
Body mass (kg)	71.4 ± 12.1	71.7 ± 11.9	70.9 ± 11.7	70.9 ± 11.6
Hematocrit (%)	44.2 ± 3.4	44.6 ± 3.3	44.2 ± 2.8	44.7 ± 2.7
Heart rate (beats∙min^−1^)	87 ± 20	85 ± 11	86 ± 17	84 ± 11
Plasma osmolality (mOsm∙kg^−1^)	288.8 ± 3.7	287.9 ± 3.7	286.4 ± 6.7	287.3 ± 5.5
Plasma aldosterone (pg∙mL^−1^)	213 ± 121	210 ± 112	215 ± 147	217 ± 119

Values are pre-exercise values presented as the means ± SD.

## Data Availability

The data will be made available from the corresponding author upon reasonable request.
